# The Present Status of Gonorrheal Infection in Women

**Published:** 1892-10

**Authors:** E. B. Jackson

**Affiliations:** Houston, Texas


### DANIEL'S TEXAS MEDICAL JOURNAL.

ESTABLISHED JULY, 1885.

PUBLISHED MONTHLY.—SUBSCRIPTION $2.00 A YEAR.

Vol. VIII. AUSTIN, OCTOBER, 1892. No. 4.

### Original Contributions.

For Daniel's Texas Medical Journal.

### THE PRESENT STATUS OF GONORRHEAL INFECTION IN WOMEN.

With Report of a Case.

E. B. JACKSON, M. D., HOUSTON, TEXAS.

THE calamitous course which this disease sometimes takes in women is becoming more generally appreciated. It has attracted a great deal of attention lately from many prominent physicians all over the country, some of whom are still devoting arduous study to the subject.

The gravity of the disorder is at once appreciated when we come to consider the deplorable pathological fields which are left along the route of the virus from the external genitals to the free ends of the tubes.

We are now acquainted with some interesting pathologies having to do with this disease, which were formerly too often supposed to be the result of some other morbid element.

Many distressing and all too intractable pelvic disorders are now recognized lamentably often as the result of gonorrheal infection.

A decade ago this was not the case. I have notes from a lecture delivered to more than a hundred gentlemen, setting forth to them that gonorrhea might be regarded as much easier of cure and less likely to produce troublesome complications in women than in men. Such a proposition we now know to be rubbish.

The female urethra suffers less, it is true, than the male, but the bladder, ureters and kidneys are no more exempt; and it is not even here that we get our most alarming cases.

Perhaps many who may read this article were not at first taught that endometritis, metritis, peritonitis with adhesions, tubal inflammation with pus, sterility and terrible dysmenorrhœa, so frequently resulted from this infection; but in the light of the present information concerning these disorders and this poison they no doubt now agree that, as a serious and lasting pelvic disease producing agent, the gonorrheal virus is entitled to as much consideration and serious study as puerperal sepsis; and here I will add that I have heard one prominent authority remark that "many of the apparently puerperal septic cases are of gonorrheal origin."

I shall not endeavor to discuss the theory or history of the gonococcus; and in drawing attention to the action and treatment of this disease in the female I shall be as brief as possible.

At first when the urethra is affected the changes which occur are practically the same as in the male, which are pain, redness, swelling etc., followed by a discharge. It is granted that these are less severe in women—hence the want of proper appreciation of the disease, formerly, when it was believed to spend its force principally upon the urethra.

The poison rarely ever remains a long period in the urethra. It seems often to begin, but rarely ever to end here; it spreads to the labial mucosa, the vulva swells, the mucous surfaces erode, which accounts for the burning of urine and for the excessive itching which is felt inside the labial fold. The vulvo-vaginal glands inflame at once, but do not abscess unless their ducts become occluded; more resistance is offered by the vagina than any other part of the genital tract, its epithelium is of a more horny character, which renders it more easily fastened upon by the virus.

It is worthy of note that a vaginitis is nearly always (in every instance in my experience) confined to young women whose vaginæ have not become hardened by frequent childbirth, or taken on the fibrous change of advanced maidenhood. As a rule, the action of the virus upon the vagina is slight. When the poison reaches the os uteri, swelling, redness and erosions occur, followed by cervicitis and a thick discharge.

The uterus is next invaded, which we detect by enlargement, general tenderness, purulent discharge and a change in the character of the catamenia. When the poison enters the tubes they inflame rapidly, and throw out an exudation which, according to its character, forms a hydro or pyo-salpynx; they seal up, thicken and lengthen.

I have seen in the Royal College of Surgeons, England, several preparations presenting these characters, many of which were successfully removed by Mr. Tait during life.

The poison which reaches the fimbriated ends of the tubes, resulting in closure, may set up peritonitis.

The swelling produced by organic inflammation may occasion it also, so that in the less active forms of peritonitis it is sometimes difficult to determine whether it is caused by pressure or by the escape of virus.

In the latter case, if the escape is free, we are soon led to suspect the true nature of the case by the violent symptoms which follow, such as great pain and aching, enormous distension and tenderness of abdomen, the exudation which soon fills up all the space around the uterus, destroying its normal swing when touched by the finger; there is great shock and prostration, all signs and expressions point to impending collapse, and the patient may rapidly expire.

The ovaries are immediately attacked by this virus when it escapes from the tubes, enormous engorgement occurs which may result in permanent enlargement, adhesions and cystic change. Abscess of the ovary may occur and is accounted for as easily as the pus formation process in the vulvo-vaginal glands, viz: The ovaries are known to contain, in many adult women, small cysts which contain fluid that soon becomes highly irritating when disturbed by the engorgement which this infection produces.

Another theory, which, as far as I am aware, is my own, is that the local circulation and absorbent dyscrasia may interfere with the normal irruption of Graaffian follicles and with the absorption by fatty degeneration of the blood clot which may have formed from a recent rupture, so that the clot, instead of disappearing to give place to a healthy corpus luteum, remains as a foreign body, just as exudations which are thrown out into Douglas' pouch become encysted by the peritoneum very often, and remain until the clot is absorbed or breaks down into pus.

The treatment of the primary infection before it has invaded the uterus is practically the same as in the male, consisting in the application of a germicide sufficiently potent to destroy the gonnoccocci, incorporated in some slightly astringent fluid directed to the cure of the mucous erosions.

Of the many remedies which may be used for this purpose, I will mention chloride of zinc and gallic acid, which I am in the habit of employing as a vaginal wash once or twice a day as the case may require, using fifteen grains [?] of each in one ounce of water. Much pain in the intervals may be avoided by packing the vagina with cotton wool well powdered with iodoform and boracic acid. In the latter stage (when the discharge becomes gleety) sulphate of zinc and acetate of lead forms a desirable injection which, if used every two or three hours, will soon check the discharge completely.

The bowels should be emptied every day with a saline purgative and a full dose of bromide of potash and soda given at bed hour. This treatment should be carried out every day until the action of the poison is checked. In the uterus, the poison can be fairly dealt with if it has not entered the tubes. An operation is necessary in which an anæsthetic will be required, the vagina is first douched, the perineum is retracted by aid of a Sims' speculum, the os is drawn down by a rat-toothed vulsellum, Heger's dilators are passed one every minute until a Playfair's probe, armed with lint, will enter the uterus; after swabbing out the endomentrium with this linted probe, a new linted probe is prepared and well soaked in strong tinct. of iodine. This is passed in and thoroughly rubbed over the entire endomentrium. Now a roll of tape, which has been soaked in iodoformed glycerine, is packed in the uterine cavity, leaving two or more inches of its tail projecting into the vagina for its easy removal, which should be accomplished in five or six hours.

This operation, if done three or four successive days, is generally successful, but if it should fail, pure carbolic acid with equal part of glycerine (½ drachm each) may be applied in the same manner, taking care, of course, to leave and to keep the os well open for the discharge of the extensive slough material.

This operation is unjustifiable and dangerous if the disease has passed beyond the uterus.

In tubal, ovarian and peritoneal infection, the principal treatment consists in supporting the powers of the body with nourishing food, tonic medicine and rest.

The avoidance of everything having the power to excite increased flow or to interfere with the return of blood from the pelvis, such as coitus, cold or hot douche, loaded rectum, etc,, should be enjoined. Whenever these dull symptoms become acute, which often occurs, poultices for relief of the pain and tension will be found of more value than any other remedy.

The means of dealing with this poison when it affects the bladder, uterus and kidneys are simple. The chief remedy is copaiba, which, if the urine is made to contain in strong solution, acts as a potent poison to the gonnoccocci. Applied directly to the germs, this drug has proven to be inert; but, on the other hand, it has been proven that the urine possesses the power of setting free its coccus destroying principle. The urine, when heavily charged with this oil, is certainly destructive to them. Four sealed capsules of the ordinary dose is usually sufficient for one day. With this treatment, it is desirable to combine also the morning saline purgative and the night bromide potion above mentioned.

REPORT OF CASE.

Mrs. M, age 21; married since February, '91. Catamenia appeared at 14 years; always regular every four weeks; loss normal; some pain but very slight. Three weeks after marriage, noticed a discharge and felt occasional pains in pelvic region, which were intensified by coitus, but this she considered the probable experience of all newly married women. Her second third and fourth monthly periods thereafter were attended by more pain than she had ever before felt; sexual congress became unbearable, and in view of the facts, her husband was advised by the physician in attendance to send her away for the summer to rest and be treated. This he did in June. She returned in September somewhat improved, but immediately the discharge became increased and all her former symptoms soon returned—for obvious reasons. The periods came on every 21 days with increased flow and pain, etc., characteristic of inflammatory action somewhere in the pelvis.

She felt continnally distressed about the change her health had undergone since marriage, and expressed to her physician the belief that her second flow after becoming a wife was an abortion, and she still holds the same opinion, as no one has attempted to disabuse her mind. Each month her distress was slightly increased. I saw her in June, '92. The symptoms just given were and are still present. Bi-manually the uterus is enlarged and very tender. Both ovaries are felt enlarged and extremely sensitive and somewhat prolapsed. There is a band-like tightness and fullness in the region of the broad ligaments which may be tubes, infiltration or adhesions. There is appreciable general fullness in the floor of the pelvis. Each fornix is distorted and the uterus is but slightly mobile. The speculum reveals erasions of the os and a purulent discharge characteristic of endometritis. She has never even partial freedom from pain except when reposing on a couch, which she is forced to keep, uninterruptedly, through each period of the menstrual nisus. Thus imprisoned from the brightness and activities of the outside world, her spirit has been supplanted by melancholy depression. No wonder attaches to the change, for it is only too well known that reflex nervousness must always come in long standing cases of pelvic inflammatory action.

This case has been related tamely and is only one of many which exist at the present moment. They belong to the serious and baffling class of disorders from which, no doubt, every humane physician would fain shrink to witness or discuss. Think of it! Is it not a terrific warning to doctors who give their consent to the marriage of a man thirty, sixty or ninety days after a specific discharge?

Among the pitiable martyrdoms which we are sometimes called upon to witness, there is probably no one worse than this. A tender, young, innocent woman, so recently a light-hearted girl, doomed eventually to abdominal section with its grave risks, or to invalidism for life. Could the inestimable blessing of preventive medicine be more forcibly argued than it is by such a case as this? But to conclude.

Many would say operate just as soon as the patient can be properly prepared for the ordeal, i. e., if the ovaries are large enough to be considered cystic and there is evident disease of the tubes. Many others would say, she is young, encourage her to be cheerful and to wait. In either case "the rub" confronts us, for we are poor comforters when we meet with difficulties insuperable to medicine; and on the other hand, our regret must be of the profoundest nature when we have to inform a confiding patient, little more than a child and crushed already with suffering, that her only hope of permanent relief lies in an operation of a serious nature. Some claim that conservatism is too frequently ignored in cases who have not lived out a goodly portion of their lives. Others ask why should a case at any age be allowed to progress until it has destroyed the recuperating powers, rendering dangerous the operation, which, if done early, they claim, by only small risks. It is appreciated that hard and fast rules can not be laid down which will answer for all cases, but it is to be hoped that a universal settlement of the vexed question may very soon obtain so that the lines of prohibition and justification may more satisfactorily be arrived at.

